# Test-Retest Reliability and Convergent Validity of Two Brief Fruit and Vegetable Intake Questionnaires among School-Aged Children

**DOI:** 10.3390/nu9070707

**Published:** 2017-07-06

**Authors:** Youjie Zhang, Marla Reicks

**Affiliations:** Department of Food Science and Nutrition, University of Minnesota, 1334 Eckles Ave., St. Paul, MN 55108-6099, USA; mreicks@umn.edu

**Keywords:** validation, assessment, survey and questionnaire, fruit, vegetable, children

## Abstract

Reliable, valid, and easy-to-implement tools are required to assess children’s fruit and vegetable intake as part of behavior change-focused nutrition education programs; however, the availability of such instruments is limited. The purpose of this study was to examine the reproducibility and accuracy of two brief fruit and vegetable intake questionnaires among 8- to 12-year-old children. A total of 109 participants from diverse racial/ethnic groups were recruited from urban afterschool programs. The results of two short questionnaires (food web and plate activity) were reproducible between two repeated measures conducted one week apart. Compared to a reference 24-h dietary recall, the food web questionnaire had acceptable convergent validity for assessing children’s fruit intake (kappa: 0.51; *r* = 0.53, *p* < 0.001), but limited validity for assessing children’s vegetable intake (kappa: 0.43; *r* = 0.33, *p* < 0.003). Children tended to overestimate intake when visualizing fruit and vegetable consumption via the plate activity questionnaire, indicating that this questionnaire was not a valid tool to assess children’s fruit and vegetable consumption at dinner meals. Children’s report of fruit intake via the food web questionnaire may be a useful indicator of program success in improving fruit intake.

## 1. Introduction

Dietary assessment methods are designed to quantify intakes of energy, nutrients, foods, and food groups as well as dietary patterns, eating habits, and overall dietary quality [1]. Generally, obtaining more dietary detail requires greater resources, participant time, and/or administrative input. For example, the 24-h dietary recall (24hDR) method obtains detailed information about all foods and beverages consumed in a day, which can be applied to describe a population’s intake or to estimate an individual’s usual intake with multiple administrations. This method usually requires a trained recall staff more than 20 min to collect an individual’s one-day consumption [2]. In comparison, a short food questionnaire regarding specific food items may be completed in a few minutes by a group of people [3]. The latter is more readily implemented in the field under constraints of limited time and resources, but accuracy may be compromised. Therefore, selecting an appropriate dietary assessment method depends on its functionality as well as feasibility.

Fruits and vegetables are key elements of healthy eating patterns [4]. Increasing the consumption of fruits and vegetables has been shown to promote weight loss and prevent chronic diseases such as coronary heart disease, type 2 diabetes, and certain types of cancer [5,6]. Many programs have been implemented to increase children’s fruit and vegetable intake with improved consumption considered a direct indicator of program success [7,8]. In addition, the MyPlate message of “make half your plate fruits and vegetables” has been widely applied to promote fruit and vegetable intake and is one of the key behavioral outcomes of the Supplemental Nutrition Assistance Program Education (SNAP-Ed) in the United States [9]. To date, no reliable, valid, and easy-to-implement instrument exists to assess school-aged children’s fruit and vegetable intake and compliance to the MyPlate message [10]. The purpose of this study was to evaluate the test-retest reliability and convergent validity of two brief fruit and vegetable questionnaires that were designed to evaluate behavioral outcomes of nutrition education programs among school-aged children (8- to 12-year-old).

## 2. Materials and Methods

### 2.1. Questionnaires

The Food Web Questionnaire (FWQ) was a one-day food record with eight fillable circles. It was adapted from a food liking questionnaire used with school-aged children who participated in SNAP-Ed sessions [11]. Participants were asked to write eight foods they consumed yesterday in the fillable circles.

The Plate Activity Questionnaire (PAQ) was designed based on the MyPlate message of “make half your plate fruits and vegetables”. It had one example photo of a dinner plate and five plate drawings with 0, 1/8, 1/4, 3/8, 1/2 of the plate shaded to represent the proportion of the plate covered by fruits and vegetables. Participants were asked to select one drawing that matched the amount of fruits and vegetables that covered their dinner plate in the previous evening. The drawing selected was regarded as a proxy for the amount of fruits and vegetables eaten at dinner and was converted to scores from 0 to 4.

Both the FWQ and PAQ were administered using standard written scripts. The PAQ was administered immediately after the FWQ. The two questionnaires took less than 10 min to complete. The FWQ and PAQ are available as supplementary materials (File S1).

### 2.2. Study Participants

All participants were recruited from five urban afterschool programs that primarily served children from low-income families in St. Paul and Minneapolis, Minnesota using flyers and verbal announcements between July and November of 2015. The inclusion criteria were being between 8 and 12 years of age and able to read, speak, and write English. A total of 115 children were recruited. Two refused to participate and four dropped out during the study. Signed child assent and parent consent forms were obtained from participants and their caregivers. Each participant was given a $10 gift card in return for participation. This study was approved by the University of Minnesota Institutional Review Board (study number: 1505P69902).

### 2.3. Demographic and Anthropometric Measurements

Participants reported their age, gender, and race/ethnicity. Height was measured using a seca 202 measuring rod (seca, Hamburg, Germany) to 0.1 cm. Weight was measured using a Tanita HD-351 digital scale (Tanita, Tokyo, Japan) to 0.01 kg. Body mass index (BMI) was calculated as body weight (kg) divided by height squared (m^2^). Children’s weight status was determined using Center for Disease Control and Prevention gender-specific BMI-for-age percentile growth charts and weight status categories [12].

### 2.4. Study Design

Participants (*n* = 27) from one urban afterschool program completed the FWQ and PAQ twice from seven to ten days. One day before the administration of the questionnaires, they also photographed their dinner at home before and after consumption using a placemat and a digital camera. Participants (*n* = 82) from four other urban afterschool programs completed the FWQ and PAQ immediately followed by a 24hDR. The 24hDR was selected as the reference measure because it has been shown to be a relatively reliable dietary assessment tool among children as young as third grade [13]. In addition, the testing measures (FWQ and PAQ) and the reference measure (24hDR) are all subject to recall error; this similarity facilitates comparison. The researcher (Y.Z.) was trained and certified to conduct 24hDRs using the Nutrition Data System for Research (NDSR) software program, 2015 version (Nutrition Coordinating Center, University of Minnesota, Minneapolis, MN, USA). Data were collected on an individual basis.

### 2.5. Data Analysis

Two research assistants independently counted numbers of fruits and vegetables and compared types of fruits and vegetables reported in the FWQ and 24hDR. Discrepancies in numbers and item-wise comparisons were resolved before statistical analysis.

Test-retest reliability was assessed at the group level because the FWQ and PAQ were sensitive to the day-to-day variation in food intake by individuals [14,15]. Test-retest reliability was indicated by a lack of significant differences in counts of fruits and vegetables and PAQ scores between repeated measures at the group level.

For the FWQ, convergent validity was assessed by percentage of agreement, weighted kappa statistics, and differences in mean or median counts of fruits and vegetables with the 24hDR, as well as by correlations between counts of fruits and vegetables based on the FWQ and servings of fruits and vegetables based on the 24hDR. In addition, convergent validity of the FWQ against the 24hDR was examined by item-wise comparisons. An item reported in both the FWQ and the 24hDR was classified as a match; an item reported in the FWQ but not in the 24hDR was classified as an intrusion; and an item reported in the 24hDR but not in the FWQ was classified as an omission.

For the PAQ, convergent validity was assessed by correlations between PAQ scores and servings of fruits and vegetables eaten at dinner as reported in the 24hDR. Three nutritional professionals independently assigned PAQ scores based on dinner plate photos with clear pre- and post-consumption food images. A final PAQ score was determined based on the agreement of at least two nutritional professionals. The validity of the PAQ was further examined by comparing the percentage of agreement, kappa statistics, and differences in mean or median of child-reported PAQ scores and researcher-assigned PAQ scores.

Wilcoxon signed rank test and Spearman correlation analysis were performed for non-parametric data. The strength of kappa statistics and the Spearman correlations were interpreted as poor (≤0.2), fair (0.21–0.40), moderate (0.41–0.60), good (0.61–0.80), or very good (0.81–1.0) [16]. Children’s reporting accuracy may be influenced by age, gender, and weight status. Univariate analysis of variance and Tukey post hoc tests were used to examine abilities of the FWQ and PAQ to rank children’s fruit and vegetable intake as well as differences in participants’ reports for the 24hDR and testing questionnaires by age, gender, and weight status. The significance level was set at *p* < 0.05. All statistical analyses were completed using SAS software (version 9.4, SAS Institute, Inc., Cary, NC, USA).

## 3. Results

### 3.1. Participant Characterstics

The total sample consisted of 109 children (53% female, 47% male) from 8 to 12 years of age; 95% were between 9 and 11 years of age. Nearly half were black (48%); the remaining were mixed race (34%), Hispanic (9%), Native American (5%), white (4%), and Asian (1%). About half (44.6%) were either overweight or obese. Children’s fruit and vegetable intakes are presented in Table 1.

### 3.2. Test-Retest Reliability

The FWQ was reliable between repeated tests, as indicated by the relatively consistent numbers of participants reporting no fruit intake (*n* = 12 and 11), two or more fruits (*n* = 7 and 5), no vegetable intake (*n* = 12 and 13), and two or more vegetables (*n* = 7 and 6) among twenty-seven participants who completed the FWQ one week apart. No significant differences were observed in terms of counts of fruits and vegetables between repeated measures at the group level (*p* = 0.95 and 0.64, respectively).

The results of testing the PAQ also indicated test-retest reliability. Similar to the FWQ, relatively consistent numbers of participants had a PAQ score ≤2 (*n* = 13 and 15) and a PAQ score >2 (*n* = 14 and 12). No significant PAQ score differences were observed between repeated measures (*p* = 0.25). About two thirds of participants changed their fruit count and vegetable count according to their FWQ and PAQ scores, which reflected the day-to-day variation in fruit and vegetable consumption.

### 3.3. Convergent Validity

**Figure 1:**
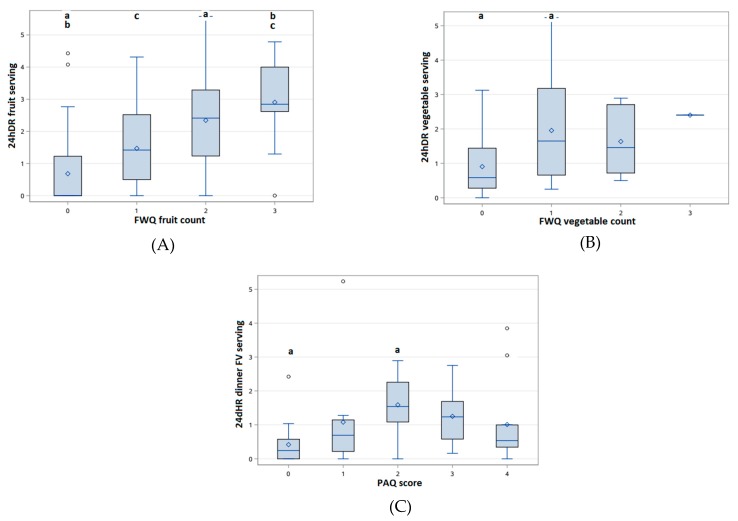
Comparison of participants’ 24hDR fruit and vegetable servings by FWQ and PAQ responses. (**A**): 24hDR fruit servings by FWQ fruit counts; (**B**): 24hDR vegetable servings by FWQ vegetable counts; (**C**): 24hDR fruit and vegetable servings at dinner by PAQ scores. Bars marked with the same letter indicated *p* < 0.05 in Tukey post hoc tests. FWQ: food web questionnaire; PAQ: plate activity questionnaire; 24hDR: 24-h dietary recall; FV: fruit and vegetable.

Table 2 shows mixed findings regarding convergent validity of the FWQ against the 24hDR. The percentage and strength of agreement were moderate for both fruit count and vegetable count. The FWQ did not yield significantly different fruit counts but showed significantly different vegetable counts compared to the 24hDR. The fruit count and vegetable count in the FWQ were correlated with servings of fruit and vegetables in the 24hDR, respectively. In addition, the FWQ was able to rank children’s fruit intake (Figure 1A) but not vegetable intake (Figure 1B). Children who reported eating two or three fruits in the FWQ reported significantly higher servings of fruit intake in the 24hDR. However, the match rate was low and omission and intrusion rates were high when types of fruits and vegetables were compared between the FWQ and the 24hDR (Table 3).

The PAQ showed poor convergent validity with both plate photos and the 24hDR. According to 36 plate photos assessed, participants were more likely to overestimate (42%) than underestimate (25%) the amount of fruits and vegetables on the dinner plate. Comparing participant-reported and researcher-assigned PAQ scores, there was a 0.69 point difference (*p* = 0.02). The strength of agreement was fair (kappa: 0.25, 95% confidence interval (CI): 0.05–0.45). In addition, participant-reported PAQ scores were correlated with fruit and vegetable servings reported at dinner time in the 24hDR (*r* = 0.24, *p* = 0.03). The PAQ also did not show ability to consistently rank children’s fruit and vegetable intake at dinner (Figure 1C). No significant differences were observed in any of the outcome variables by gender, age, or weight status.

## 4. Discussion

The current study examined the performance of the FWQ and PAQ, two short instruments designed for assessing the consumption of fruits and vegetables among 8- to 12-year-old children participating in nutrition education programs. Both instruments exhibited test-retest reliability when administered one week apart. The FWQ showed partial validity in counting and ranking children’s fruit intake but not vegetable intake. Golley and colleagues proposed three criteria for interpreting the validity of short food intake questionnaires: (1) *p* > 0.05 for *t*-tests; (2) *r* > 0.5 for Pearson or Spearman correlations; and (3) kappa > 0.6 between testing and reference methods [10]. Considering all three criteria, the FWQ showed partial adequacy for counting children’s fruit intake (*p* > 0.05 and *r* = 0.53); and inadequacy for counting children’s vegetable intake.

While the FWQ produced mixed results regarding adequacy for counting fruit and vegetable intake, it demonstrated comparable validity with other validated short instruments measuring fruit and vegetable intake among children of a similar age group. A fruit and vegetable food frequency questionnaire asked third graders “In the past month, about how many servings of fruits/vegetables did you eat?” with response options ranging from “never” to “five or more times per day” [17]. This instrument showed low validity (*r* < 0.3) against seven-day food records [17]. The School Physical Activity and Nutrition questionnaire (SPAN) collected fourth graders’ responses to “Yesterday, did you eat any fruits/vegetables?” with options ranging from “no” to “yes, three or more times yesterday” [18]. This questionnaire was also validated against the 24hDR. Compared to the FWQ, the SPAN had a relatively lower percentage of agreement for vegetable count (27%), weaker strength of agreement for fruit count (*k* = 0.27) and vegetable count (*k* = 0.17), and weaker correlation of fruit count (*r* = 0.40) with 24hDR. Remembering and conceptualizing frequencies of food intake or habitual food intake are challenging for 8- to 12-year-old children [19]. How they cognitively process these types of questions remains unclear. 

The FWQ instructed children to write down names of foods they consumed, which may allow for less long-term memory decay and avoidance of the challenge of abstract conceptualization. Adopting a similar approach, the Day in the Life Questionnaire (DILQ) provided chronologically-ordered instructions for children to complete a food intake and physical activity diary [14]. This instrument showed good agreement between lunch-time fruit and vegetable intake (kappa > 0.6) and school lunch observations among third and fourth graders [20]. The FWQ was easy to implement and required less time (10 min) than the DILQ (30 min). The Tukey post hoc test also confirmed the acceptable performance of the FWQ in ranking children’s fruit intake. When time is limited, the FWQ can serve as an adequate or better alternative to the DILQ because the DILQ showed poor validity for quantifying all day fruit and vegetable intake when validated against 24hDR among third graders (*r* = 0.16 for fruits and *r* = 0.23 for vegetables) [21]. However, comparisons of results across studies should be considered with caution because there may be considerable variations in participants’ demographic characteristics, study design, and instrument administration. Further analysis of item-wise comparisons for the FWQ showed interesting but problematic findings based on low match rates and high omission and intrusion rates at the group level. Better match rates (>60%) and lower omission and intrusion rates (<20%) have been observed in previous studies using additional aids (food diary and school menu) in food recalls [22,23]. The current study did not apply these strategies to assist participants in recalling intake. The low item-wise agreements between the FWQ and 24hDR indicate that children of this age range have limited ability to recall types of foods they consume sporadically. If the food intake information needs to be precise, additional strategies to enhance recall may be necessary. Reducing the retention interval by asking children to write down food eaten in the same day or providing school menus or pictures of common foods may help improve recall accuracy.

The overestimation of fruit and vegetable intake is a reoccurring theme in the validation of child-reported food intake measurements [17,21,24,25,26]. Participant-provided plate photos shed light on the difficulty of estimating proportions of fruits and vegetables on dinner plates. The plate photos showed that a number of dinner meals were not served on plates or that vegetables were served within mixed dishes, making it difficult to determine the identity of ingredients. The finding that children tend to overestimate the portion size of fruits and vegetables indicates the need to address this issue while delivering the message of “making half of the plate fruits and vegetables”.

The current study had several limitations. U.S. national dietary intake data showed that about 41% of children’s vegetable intake came from mixed dishes [27]. The FWQ was designed to count whole foods only, which may contribute to the inadequate validity of quantifying children’s vegetable intake. In addition, some children consumed more than one of a certain type of fruit or vegetable, such as two oranges; but only wrote this food down once. This may also impair the sensitivity of the FWQ in quantifying children’s intake. Due to the logistic constraints, the FWQ and PAQ were conducted with one child at a time, which was different from the intended design of group administration. When conducting the FWQ with more than one child, those administering the questionnaire need to closely monitor the progress, provide help in spelling, and check the quality of completion. In addition, both the 24hDR and the testing questionnaires are subjective measures of dietary intake. Despite that the similarity facilitates comparisons for convergent validity; these measures may not fully capture participants’ actual intake.

## 5. Conclusions

The FWQ is a useful tool for ranking children’s fruit intake with acceptable accuracy. An increased rate of children reporting two or more fruits in the FWQ may be considered an indicator of program success in improving children’s fruit intake. However, the FWQ was not a valid tool for ranking children’s vegetable intake, and the use of the PAQ was also discouraged due to its poor validity and ranking capability.

## Figures and Tables

**Table 1:** Fruit and vegetable consumption among participants measured by the FWQ and 24hDR (*n* = 82).

	FWQ (Count)			24hDR (Count)			24hDR (Serving)
	Mean	Median	Range	Mean	Median	Range	Mean ± SD
Fruit	1.0	1.0	0–3	1.3	1.0	0–5	2.0 ± 2.4
Vegetable	0.8	1.0	0–6	1.2	1.0	0–10	1.8 ± 2.1

FWQ: food web questionnaire; 24hDR: 24-h dietary recall; SD: standard deviation.

**Table 2:** Comparisons between the FWQ and 24hDR by counts of fruits and vegetables among 8 to 12 years old (*n* = 82).

	% Agreement	Kappa (95% CI)	Wilcoxon Signed Rank	Spearman Correlation
**Fruit**	51	0.51 (0.37, 0.65)	*p* = 0.06	0.53, *p* < 0.001
**Vegetable**	59	0.43 (0.26, 0.60)	*p* = 0.006	0.33, *p* < 0.003

FWQ: food web questionnaire; 24hDR: 24-h dietary recall; CI: confidence interval.

**Table 3:** Match, omission and intrusion rates of types of fruits and vegetables reported in the FWQ vs. 24hDR (*n* = 82).

	Match % ^a^	Omission % ^b^	Intrusion % ^c^
Fruit	38.4	37.0	24.6
Vegetable	37.2	44.2	18.6

^a^ Match % = matches × 100/(24hDR + intrusions); ^b^ Omission % = omissions × 100/(24hDR + intrusions); ^c^ Intrusion % = intrusions × 100/(24hDR + intrusions).

## Supplementary Materials

The following are available online at www.mdpi.com/2072-6643/9/7/707/s1, File S1: The food web questionnaire and the plate activity questionnaire with instructions.
