# Supplementary material for: Test-Retest Reliability and Convergent Validity of Two Brief Fruit and Vegetable Intake Questionnaires among School-Aged Children
Source: Nutrients. 2017 Jul 6;9(7):707. doi: 10.3390/nu9070707 (PMC5537822; doi:10.3390/nu9070707)
Supplement: Supplementary file 1 [file nutrients-09-00707-s001.zip › nutrients-203049-supplementary.pdf]

File S1: The food web questionnaire and the plate activity questionnaire with instructions.

Instructions

1. Food Web Questionnaire

People eat many different kinds of foods. Please take a minute to think about what kinds of food you ate yesterday.

Now I would like you to write down the foods you ATE yesterday, from midnight to midnight, in the bubbles on the sheet. Please write one food in each bubble. I want you to be as clear as you can. For example, instead of saying "Chips" write "potato chips" or "corn chips". Instead of saying "fruits", write "banana" or "apple". If you ate at a restaurant, write the name of the food.

Do you have any questions?

2. Plate Activity

Now that you have written down the foods that you ate yesterday, please take a minute to think about what you ate for dinner last night.

After you think about what you ate, I would like you to think about how much of your dinner plate was filled with fruits and vegetables. There is a picture that helps you do this. If you had fruits and vegetables which were not on your big plate, remember to include those as a part of your big plate. Then, check the box below the plate which matches best with the amount of your plate covered with fruits and vegetables. You can only check one box.

Student ID: ___________ Site: ___________ Researcher ID: __________

Please write ONE FOOD in each bubble.

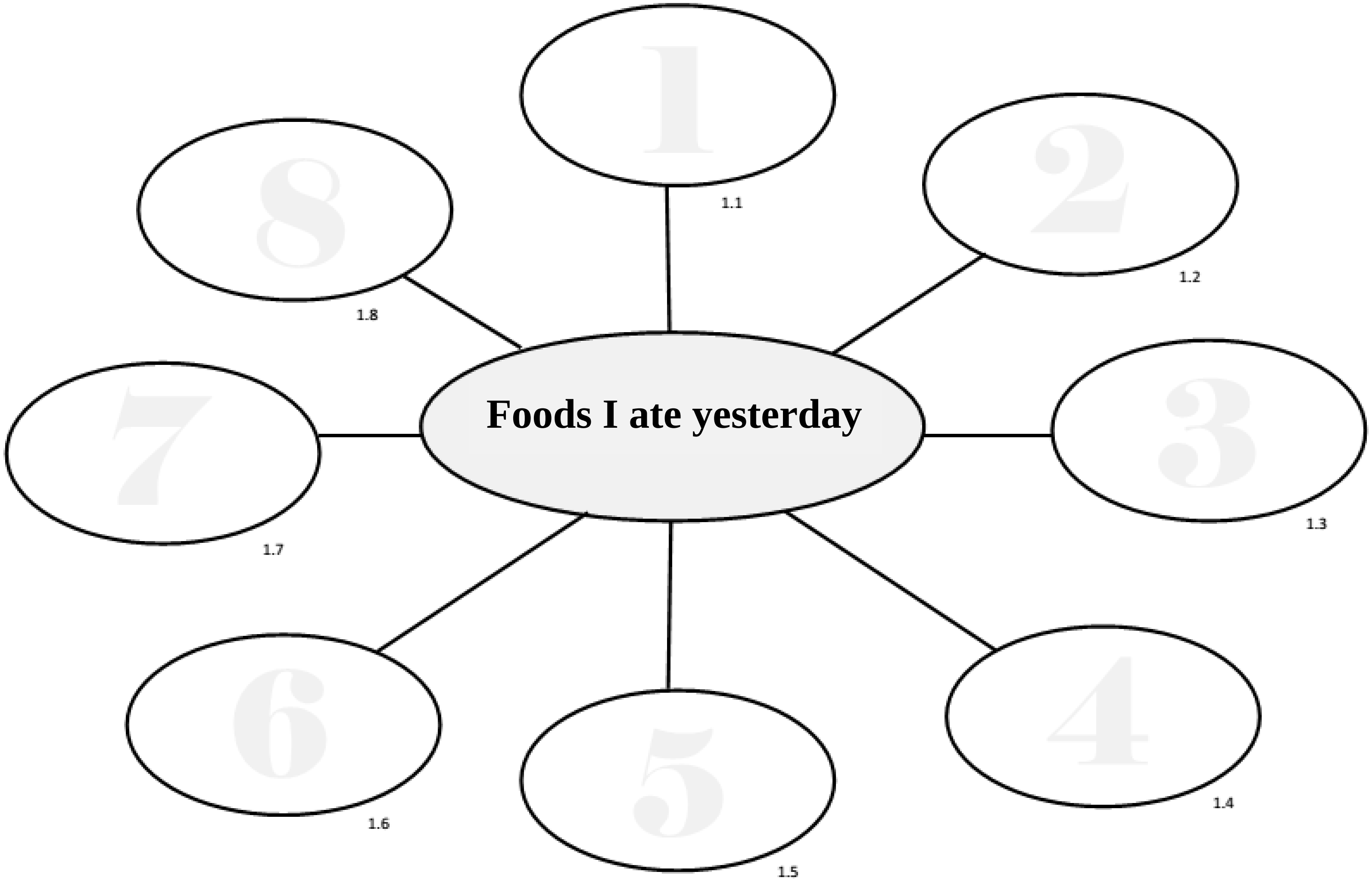

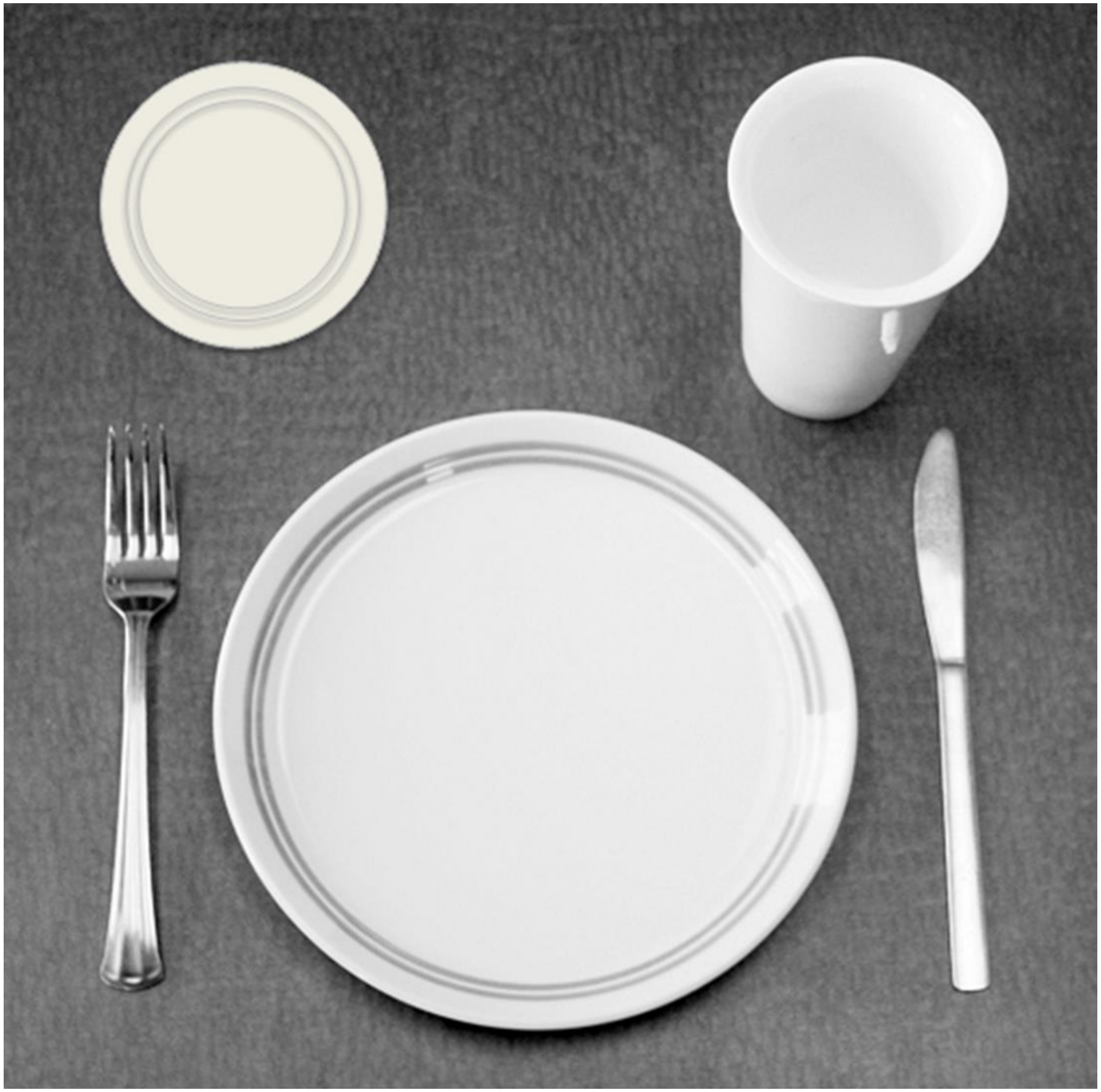

2.1 The SHADED AREA shows how much of your dinner plate was filled with fruits and vegetables last night. Please check the box below the ONE PLATE which is the best match.

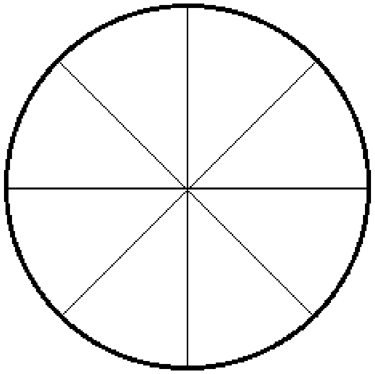

☐ None

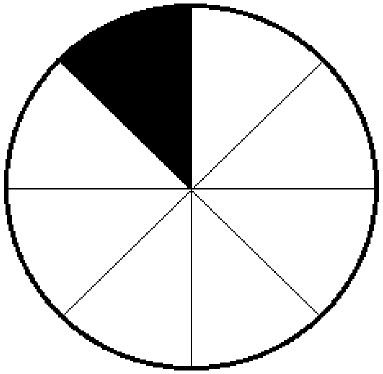

☐ 1/8

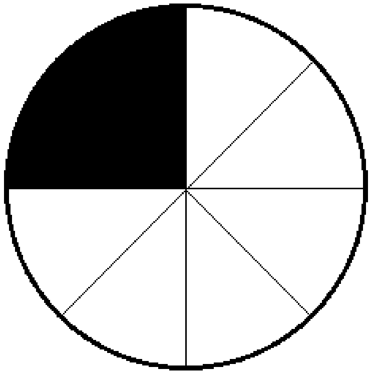

☐ 1/4

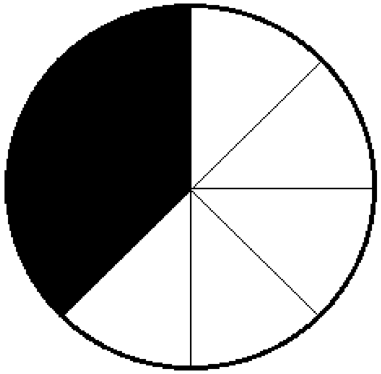

☐ 3/8

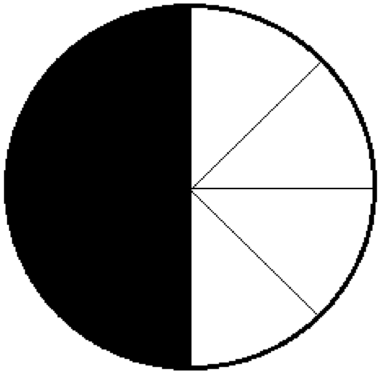

☐ 1/2
